# Bone Regeneration and Polyetheretherketone Implants in Maxillo-Facial Surgery and Neurosurgery: A Multidisciplinary Study

**DOI:** 10.3390/biology13070467

**Published:** 2024-06-25

**Authors:** Mattia Todaro, Gianmarco Saponaro, Federico Perquoti, Giulio Gasparini, Francesco Signorelli, Tommaso Tartaglione, Alessandro Moro

**Affiliations:** 1 Maxillo Facial Surgery Unit, Fondazione Policlinico Agostino Gemelli IRCCS Hospital, 8 Largo Agostino Gemelli, 00168 Rome, Italy; mattia.todaro@policlinicogemelli.it (M.T.); federico.perquoti01@icatt.it (F.P.); giulio.gasparini@policlinicogemelli.it (G.G.); alessandro.moro@policlinicogemelli.it (A.M.); 2 Neurosurgery Unit, Fondazione Policlinico Agostino Gemelli IRCCS Hospital, 8 Largo Agostino Gemelli, 00168 Rome, Italy; francesco.signorelli@policlinicogemelli.it; 3 Radiology Department, Fondazione Policlinico Agostino Gemelli IRCCS Hospital, 8 Largo Agostino Gemelli, 00168 Rome, Italy; tommaso.tartaglione@policlinicogemelli.it

**Keywords:** cranioplasty, maxillo-facial reconstruction, PEEK prosthesis, reconstructive surgery

## Abstract

**Simple Summary:**

In this study, we explored the use of polyetheretherketone (PEEK) implants in maxillofacial and neurosurgery, focusing on bone regeneration and implant outcomes. We investigated the effectiveness of PEEK implants in reconstructing craniofacial defects caused by trauma, tumor removal, or congenital malformations. Through analysis of postoperative CT scans, we observed significant bone regrowth around PEEK implants in most cases, indicating successful integration with surrounding tissue. However, we also identified instances of bone resorption in some patients, highlighting potential complications that need further attention. Our findings underscore the importance of optimizing the interface between implants and bone to ensure long-term stability and patient outcomes. Overall, this research contributes valuable insights into the use of PEEK implants in surgical procedures, potentially improving treatments and enhancing patient quality of life.

**Abstract:**

Polyetheretherketone (PEEK) in the last few years has emerged as an exceedingly promising material for craniofacial defects due to its biocompatibility and mechanical properties. However, its utilization remains controversial due to its inertness and low osteoinductivity. This study aimed to investigate the postoperative outcomes of patients undergoing maxillo-facial and neurosurgical procedures with PEEK implants. The focus is on evaluating bone regrowth on the surface and edges of the implant, periosteal reactions, and implant positioning. A retrospective analysis of 12 maxillo-facial surgery patients and 10 neurosurgery patients who received PEEK implants was conducted. CT scans performed at least one year post operation were examined for bone regrowth, periosteal reactions, and implant positioning. In maxillo-facial cases, the analysis included mandibular angle and fronto-orbital reconstruction, while neurosurgical cases involved cranioplasty. In maxillofacial surgery, 11 out of 12 patients showed radiological evidence of bone regrowth around PEEK implants, with favorable outcomes observed in craniofacial reconstruction. In neurosurgery, 9 out of 10 patients exhibited minimal or none bone regrowth, while one case demonstrated notable bone regeneration beneath the PEEK implant interface. The study highlights the importance of implant design and patient-specific factors in achieving successful outcomes, providing valuable insights for future implant-based procedures.

## 1. Introduction

Reconstruction of extensive defects in head and neck—resulting from trauma, oncologic resection, and malformation—is a demanding challenge for maxillofacial surgeons and neurosurgeons. In this context, the role of surgeons becomes crucial in providing innovative and personalized solutions to enhance the functional and cosmetic outcomes of patients. Over the past two decades, numerous autogenous and alloplastic materials have been utilized in maxillo-facial surgery and neurosurgery, with both positive and negative aspects for each material. The ongoing research in craniofacial surgery underlines the use of new biomaterials and surgical instruments, and PEEK and other technologies and materials are included in this effort [1,2,3].

PEEK, a member of the polyaryletherketone (PAEK) family, is a semi-crystalline linear polycyclic aromatic thermoplastic first developed in England in 1978 [4]. It quickly became an excellent substitute for replacing metal implant components in orthopedic and traumatic surgery, eliminating concerns regarding metal allergies, and offering excellent mechanical and biological qualities [5].

Despite its rigid aromatic molecular backbone, unfilled PEEK exhibits significant ductility and can withstand substantial plastic deformation under both uniaxial tension and compression. Due to its elastic modulus, PEEK offers a mechanical structure close to that of human cortical bone and remains relatively unaffected by temperature variations at body temperature [6,7].

The stable chemical structure makes PEEK biologically inert, offering excellent resistance to chemical and thermal degradation [8] and remaining solid and unaffected by sterilization [9]. In addition, PEEK exhibits good biocompatibility in vitro [10] and in vivo [11], causing neither toxic or mutagenic effects nor clinically significant inflammation.

However, its relative inertness in a biological context [12] has also limited its potential applications. Enhancing the biomechanical properties of PEEK to improve osteointegration, ensure long-term implant stability, and control implant infections are the primary objectives driving current research efforts. In these terms, physical and chemical treatments are two main strategies utilized over the past decade to enhance the bioactivity of PEEK [13].

Despite the considerable volume of published literature on PEEK implants, there is a lack of multidisciplinary postoperative studies proposing personalized modalities for maximizing its utilization and describing different techniques that may have long-term impacts. Moreover, while the use of PEEK has been described in both specialties and new material modification methods are being experimented, the number of described patients remains limited [14]. This study aims to investigate the postoperative outcomes of patients undergoing maxillofacial and neurosurgical procedures with PEEK implants, focusing on evaluating bone regrowth on the implant surface and edges, periosteal reactions, and implant positioning.

### 1.1. PEEK Applications in Neurosurgery

Head injuries, cerebral tumors, ischemia, and infections commonly lead to intracranial disorders, often requiring decompressive craniectomy followed by reconstruction [15,16]. The first reported PEEK cranioplasty was in 2007, aiming to find a material with the ability to promote growth, resist infection, and be readily available [17].

In addition to cranial reconstructions, PEEK implants find utility in spinal surgery. They are commonly employed in interbody fusion procedures, where they serve as cages or spacers to restore disc height and stabilize the spine [18]. PEEK’s radiolucency enables accurate assessment of fusion progression, while its biomechanical characteristics promote osseointegration and minimize stress shielding [19].

Today, computer-designed prefabricated implants can be precisely tailored to complex craniofacial defects. In this approach, CT data are utilized to create a rapid 3D prototype model using additive technologies like stereolithography or fused deposition modeling, or subtractive techniques like computer numerical control milling. Besides providing a better fit, these technologies are timesaving compared to classic impression techniques. Additionally, 3D modeling during the design phase allows for correcting trophic defects commonly found in the temporal bone [20].

Overall, our hospital admits over 400 patients annually with traumatic brain injury, approximately 40 of whom undergo decompressive craniectomy, with 30 requiring maxillofacial reconstruction. Despite autologous bone flap repositioning remaining the gold standard for survivors, around 15 cases per year undergo PEEK cranioplasty at the Department of Neurosurgery.

### 1.2. PEEK Applications in Maxillo-Facial Surgery

Reports of the use of PEEK in the reconstruction of maxillofacial defects have become increasingly frequent in the last 10 years [21,22,23]. This is because the prefabrication process that can produce patient-specific implants (PSIs) satisfies many of the requirements of maxillofacial surgery, including production speed, ease of use, and precision. In this field, CAD/CAM technologies enable manufacturing of very precise implants with complex morphology. One of the first clinical cases for application of PEEK LT1 material in cranio-facial reconstruction was reported by Scolozzi et al. in 2007 with a complex orbito-fronto-temporal reconstruction using computer-designed PEEK [24]. In the following years, several studies dealt with the use of PEEK in the maxillo-facial district.

Today, the versatility of PEEK (polyetheretherketone) extends across all realms of maxillofacial surgery, revolutionizing various procedures. PEEK prostheses have become pivotal in craniofacial reconstruction surgeries, addressing challenges posed by trauma, tumor resection, and congenital deformities [25]. Moreover, in temporomandibular joint (TMJ) reconstruction, PEEK implants are increasingly preferred to either replace or reinforce damaged joint components [26,27]. When faced with midface deficiencies or asymmetry, surgeons now consider PEEK implants an excellent material for effectively augmenting or reshaping the facial skeleton, yielding outstanding functional and aesthetic outcomes [28,29].

Facilitating the creation of facial prosthetics, PEEK plays a crucial role in crafting orbital, nasal, and auricular implants [30]. Furthermore, the rising popularity of PEEK-based dental implants stems from their commendable biological compatibility and mechanical resilience. In intricate soft tissue reconstruction procedures involving the oral cavity or facial regions, PEEK meshes or plates emerge as reliable solutions, offering both structural support and seamless contouring [31]. This widespread adoption of PEEK signifies a remarkable advancement in the field of maxillofacial surgery, promising improved patient outcomes and enhanced surgical techniques.

Over the past decade, at the Department of Maxillofacial Surgery in our hospital, we have treated more than 30 patients utilizing PEEK implants in an onlay configuration for refinement following volumetric reconstruction. Despite the higher cost compared to autologous bone reconstruction, this procedure significantly reduced the risk of surgical site morbidity while still yielding favorable aesthetic and functional outcomes.

## 2. Materials and Methods

### 2.1. Neurosurgery

We were able to retrieve 10 CT scans of 10 different patients performed at least one year after the operation. All patients underwent cranioplasty with a patient-specific PEEK implant at the Department of Neurosurgery. The decision to opt for PEEK cranioplasty was determined by the complexity of the defect after assessing the radiologic data.

Inclusion criteria were as follows:

1. PEEK OPTIMA^®^ (Hillhouse International, Thornton-Cleveleys, UK) positioning with TI screw. CT datasets were exchanged in Digital Imaging and Communications in Medicine (DICOM) format with the manufacturer firm (Synthes^®^ Warsaw, IN, USA).
2. CAD–CAM-designed prosthesis.
3. CT not newer than 1 year postoperatively; CT scans with preset parameters aimed at achieving optimal reconstructive outcomes: matrix 512 × 512, slice thickness 1.0 mm, feed per rotation 1.0 mm, reconstructed slice increment 1.0 mm, reconstructed algorithm bone, gantry tilt 0.

The collected data included patient demographics, diagnosis, medical records, operative reports, imaging studies, follow-up, and discharge data.

The study includes patients who were treated in our hospital from 2017 to 2023; 5 patients were excluded from the study for the following reasons: death and incomplete medical records necessary for the study. The average follow-up time was 1.4 years ranging from 1.1 years to 3 years (σ 0.475).

We analyzed CT scan images to evaluate the following:

- The radiologic bone reaction under the implant, specifically the periosteum on interface between the two;
- The bone reaction on the surface and on the edges of the PEEK implant;
- The position of the implants and their eventual shift.

To uniform the study outcomes, all the CT scan images were reviewed by the same radiologist.

### 2.2. Maxillo-Facial Surgery

We were able to retrieve 12 CT scans of 12 different patients performed at least one year after the operation.

All patients were affected by facial asymmetry with a hard tissue component but in the absence of occlusal problems or in which occlusal problems had been resolved by a previous orthognathic procedure.

Inclusion criteria were as follows:

1. PEEK OPTIMA^®^ (Hillhouse International, Thornton-Cleveleys, UK) positioning with TI screw. CT datasets were exchanged in Digital Imaging and Communications in Medicine (DICOM) format with the manufacturer firm (Synthes^®^ Warsaw, IN, USA).
2. CAD–CAM-designed prosthesis
3. CT not newer than 1 year from the operation; CT scans with preset parameters aimed at achieving optimal reconstructive outcomes: matrix 512 × 512, slice thickness 1.0 mm, feed per rotation 1.0 mm, reconstructed slice increment 1.0 mm, reconstructed algorithm bone, gantry tilt 0.

The collected data included patient demographics, diagnosis, medical records, operative reports, imaging studies, follow-up, and discharge data.

In our cohort of patients, five underwent mandibular angle reconstruction, and seven underwent fronto-orbital reconstruction. Among those subjects, five were affected by hemifacial microsomia sequalae, two were affected by trauma sequelae, three by plagiocephaly sequelae, and the other two demonstrated some degree of mandibular imbalance in the absence of any syndromic disease diagnosis.

The study includes patients who were treated in our hospital from 2017 to 2023; 2 patients were excluded from the study because of missing crucial information necessary for the study. The average follow-up time was 1.7 years ranging from 1.2 years to 4 years (σ 0.70).

We analyzed CT scan images to evaluate the following:

- The radiologic bone reaction under the implant, specifically the periosteum on interface between the two;
- The bone reaction on the surface and on the edges of the PEEK implant;
- The position of the implants and their eventual shift;

To uniform the study outcomes, all the CT scan images were reviewed by the same radiologist.

## 3. Results

### 3.1. Neurosurgery

Postoperative CT scan of 10 patients harboring PEEK cranioplasty were studied. Six patients had a right and four a left fronto-temporo-parietal cranioplasty.

**Figure 1:**
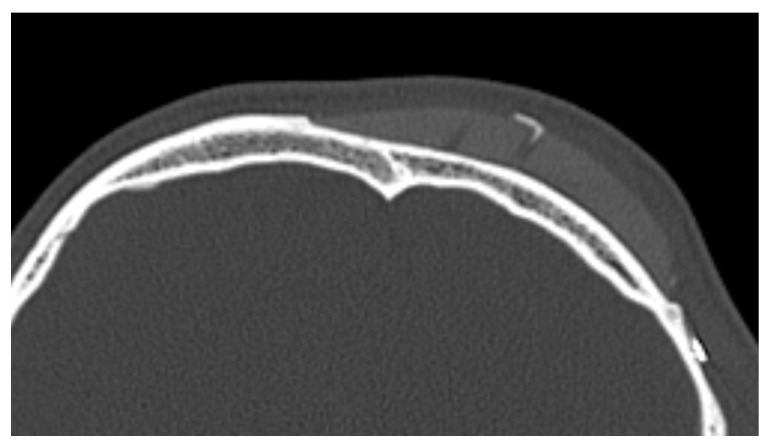
Bone formation in factory created holes of PEEK prosthesis.

In 9 out of 10 CT scans, radiological evidence of bone regrowth on the surface and edges of the implant was not evident. In one case, at 6 and 12 months, a bone regrowth consisting of islands of skull regeneration beneath the PEEK implant at the bone–implant interface was documented. This was the case of a 34-year-old female patient with a history of head injury who underwent autologous bone flap removal for infection along with wound dehiscence and secondary implant of PEEK defective cranioplasty. A retrospective analysis of the radiological exams revealed periosteal residual “spots” in the context of the defect, frontally. After PEEK cranioplasty, these spots expanded and fused to form a flake “regeneration front” of 25 mm length and 2.3 mm maximum thickness starting from the PEEK–frontal bone interface [see Figure 1].

### 3.2. Maxillo-Facial Surgery

Postoperative CT scans of 11 patients were studied. In 11 out of 12 CT scans, it was possible to note radiological evidence of bone regrowth on the surface and edges of the implant; in those implants where factory-created holes were present, bone regrowth was also noted in them.

The one case in which bone regrowth was not noticeable is a female patient where complex mandibular implants were designed with a very small bone/implant interface. No signs of bone resorption were noted at the bone/implant interface.

## 4. Discussion

PEEKs are a family of linear aromatic polymers containing ether and ketone linkages. The PEEK polymer is a semicrystalline material, classified as a high-performance engineering thermoplastic material, that has been implemented for several uses, especially in place of other metal, ceramic, and plastic implants. The PEEK material has gained popularity based on its characteristics, including strength, durability, resistance to environmental effects, and lower infection rate [32]. PEEK implants can be designed as patient-specific implants and can be planned by using patients’ CT scans. The more commonly used technique usually consists of mirroring the non-affected side on the affected side. After that, the volume and shape of the implant can then be set, and further adjustments can be made before 3D printing, to perfectly fit the implant on the recipient bone surface.

No specific implant-related complications have been reported in this field, but general complications such as infection and pain may occur [33].

PEEK prostheses are nonmagnetic and translucent to X-rays, allowing excellent visualization and study through high-resolution computed tomography (HRCT) of the prosthetic material and periosteal reaction. From the literature analysis, it emerges that the osteoconductive properties of PEEK material vary depending on factors such as surface modification, implant design, and the local microenvironment [34,35]. Its ability to promote bone growth remains a critical aspect and an open debate, within which our multidisciplinary study aims to contribute with additional data and a clearer perspective.

### 4.1. Prostheses Can Lead to Underlying Bone Resorption

Even though PEEK material was chosen as an alternative to autologous flap and ceramic hydroxyapatite implants [36,37,38], which were previously widely used but carried a high risk of infection and bone resorption, PEEK can also lead to such complications.

**Figure 2:**
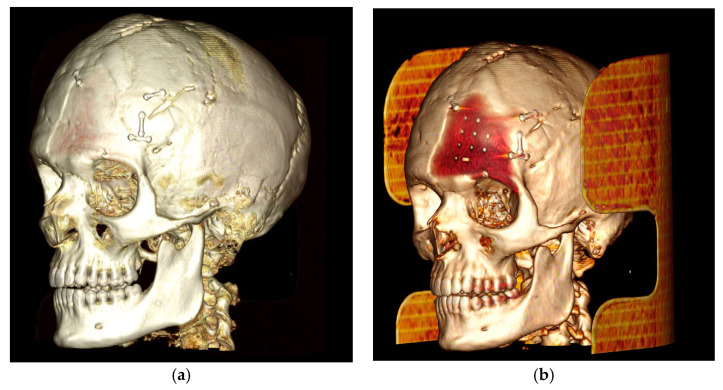
The neurosurgery case representing bone resorption. (**a**): Lateral 3D view, loaded with low volume rendering; (**b**) lateral 3D view, loaded with high volume rendering focusing on the PEEK prothesis and bone resorption underneath the prosthesis.

The case of neurosurgery underscores the potential of PEEK prosthetic implants to cause bone resorption in surrounding tissue (see Figure 2). In this instance, the observed bone resorption was minimal and did not require further intervention. Factors influencing this phenomenon include not only the material but also the design, mechanical loading, and notably, the interface between the implant and the periosteum. Titanium implants, while studies comparing different materials for craniofacial reconstruction are still limited, appear to be associated with increased donor site comorbidity, including bone resorption [39]. Countless studies have demonstrated that implants crafted from specific materials, such as titanium alloys, may induce peri-implant bone resorption attributable to stress shielding phenomena [40,41]. Stress shielding is a biomechanical phenomenon that leads to adaptive changes in bone strength due to the altered distribution of physiological loads on the bone, potentially leading to implant loosening. The elastic modulus of PEEK is comparable to those of cortical bone, so it exhibits less stress shielding than the Ti material [42]. Modifying PEEK through the addition of other materials, such as carbon fibers, is one of the techniques aimed at improving these effects, as observed in our case [43,44]. As mentioned previously, mechanical loading also plays a crucial role in potential bone resorption. Several studies have demonstrated that the mechanical behavior of implants under impact loading depends on the mechanical characterization of individual human tissues and regions [45]. This can influence the observation of bone resorption, as seen in our neurosurgical case. Finally, the micromotion of the implant, particularly observed in dental implants, could play a role in bone resorption and help explain the case observed in neurosurgery. Therefore, careful consideration of these factors is crucial in mitigating bone resorption and ensuring the long-term stability of prosthetic implants.

### 4.2. Importance of Adequate Bone–Prosthesis Interface for Osteointegration

One of the key requirements for an implant is osteointegration with the adjacent bone, allowing it to effectively transfer loads as a cohesive unit. Loss of osseointegration can result in implant loosening, leading to surgical revision and, in severe cases, implant failure [46].

**Figure 3:**
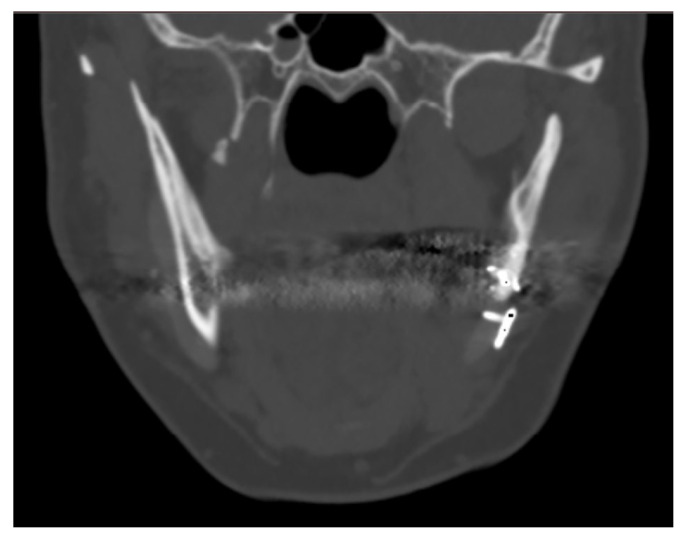
The maxillo-facial surgery case representing the absence of ossification, caused by poor interface between bone and prosthesis.

The case of maxillofacial surgery where bone regrowth was not observable underscores the importance of a sufficient bone–prosthesis interface for a successful osteointegration (see Figure 3). The neurosurgical case series, in which the interface between bone and prosthesis for cranioplasty is minimal, further corroborated this statement. Studies have shown that a larger contact area between the implant and bone facilitates osteoblastic activity and bone ingrowth [47]. In addition, implants with roughened surfaces or porous coatings promote greater bone ingrowth compared to smooth-surfaced implants [48,49]. For this reason, factory-created holes are a valuable aid in the creation of bone tissue within them, as demonstrated in the previously analyzed case. Other studies aimed at enhancing the bioactivity of PEEK to improve the bone–implant interface are still in the in vitro stage, but we anticipate significant progress soon [50,51].

Finally, the implant surface topography plays an important role in enhancing osseointegration. Cases where osteointegration has been observed predominantly occur in maxillofacial surgery, and this is not random but rather rational due to the greater bone–prosthesis interface in these procedures. Thus, optimizing the design and surface characteristics of prosthetic implants to maximize bone–prosthesis interface is essential for achieving successful osteointegration.

### 4.3. Importance of Intact and Fully Represented Periosteum

**Figure 4:**
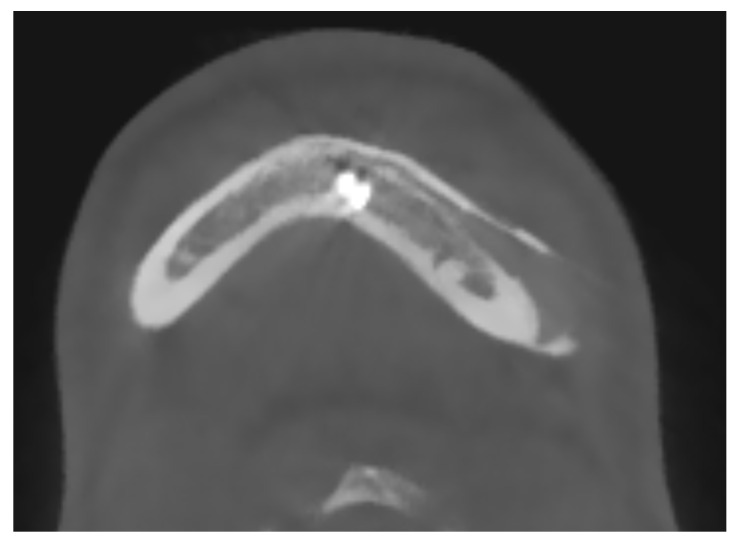
A maxillo-facial surgery case: bone regeneration around the prosthesis at the level of the left mandibular angle.

The integrity and representation of the periosteum are crucial factors in facilitating osseointegration and preventing complications associated with prosthetic implants. Indeed, regarding the periosteum reaction, what emerges from the HRCT is a non-radiologically appreciable alteration of the bone-periosteum profile, and this is a valuable sign of inert reaction of the periosteum to this material. The periosteum plays a vital role in bone healing and regeneration processes by providing a source of osteoprogenitor cells and growth factors. Preservation of the periosteum during implant surgery leads to enhanced bone formation and implant stability [52] [see Figure 4]. Conversely, damage or inadequate representation of the periosteum can impede osseointegration and increase the risk of implant failure.

From a strictly neurosurgical standpoint, three potential factors may contribute to spontaneous bone formation following large calvaria defects: the pericranium/periosteum, diploe, and dura mater [53]. Skull fractures may activate dormant osteoblasts within the periosteum and on both sides of the defect, thereby augmenting bone formation [54]. Dura mater and pericranium/periosteum may induce osteogenesis upon contact with the graft, as evidenced in rabbit studies [55]. Nonetheless, these theories are primarily grounded in animal experimentation, and there is a dearth of pertinent human research. In our scenario, the presence of PEEK implants might have played a role in fostering a phenomenon akin to the ossification wavefront of pericranial micro-islands.

In conclusion, by addressing factors such as bone resorption, bone–prosthesis interface, and periosteal integrity, clinicians can enhance the success rates of prosthetic implantation procedures and improve patient quality of life. Further research and advancements in implant design and surgical techniques are warranted to continue improving osseointegration outcomes in orthopedic surgery.

## 5. Conclusions and Limitations of the Study

The utilization of PEEK prosthetic implants presents a promising avenue in craniofacial and neuro surgeries, offering numerous advantages over traditional materials such as metals and ceramics. However, our discussion highlights the importance of acknowledging potential complications, notably bone resorption, which can possibly arise due to various factors including implant design, mechanical loading, and the interface with the periosteum. While PEEK exhibits properties conducive to osseointegration and reduced stress shielding compared to titanium, optimizing the bone–prosthesis interface remains paramount for long-term stability and patient outcomes.

Moreover, preserving the integrity of the periosteum emerges as a critical consideration for successful osseointegration, underscoring its role in bone healing and regeneration processes. Radiological assessments further indicate the importance of maintaining an inert periosteal reaction to the implant material, emphasizing the significance of intact periosteal representation. The periosteum plays a crucial role not only in the immediate response to implantation but also in the long-term success of bone regeneration, influencing the quality and rate of bone formation around the implant. It is essential to recognize that the periosteum’s ability to support the implants is integral to the bone regeneration process. Enhancing the interaction between the periosteum and the implant can potentially mitigate bone resorption and promote more robust bone growth, leading to better integration and durability of the prosthesis.

The primary limitation of the study was the variability in follow-up times. This variability arose due to differences in individual health conditions, which necessitated different monitoring, and the lack of CT scans interpreted by the same radiologist.

In conclusion, by addressing the key factors mentioned above and advancing implant design and surgical techniques, clinicians can strive to enhance osseointegration outcomes, ultimately improving patient quality of life. Continued research efforts are essential to refine our understanding and refine practices in maxillofacial surgery and neurosurgery, ensuring the continued evolution and optimization of prosthetic implantation procedures. This ongoing research is vital for developing strategies that further harness the regenerative capabilities of bone tissue, aiming for improved patient outcomes and reduced complication rates.

## Data Availability

The data are contained within the article.
